# Fungal Orbital Infection Mimicking Malignancy in a Girl

**Published:** 2015-05-01

**Authors:** Shokouh Taghipour Zahir, Naser Sefidrokh Sharahjin, Koorosh Rahmani

**Affiliations:** 1 Department of Pathology, Shahid Sadoughi University of Medical Sciences, Yazd, Iran; 2 Department of Radiology, Shahid Sadoughi University of Medical Sciences, Yazd, Iran; 3 Medical School, Shahid Sadoughi University of Medical Sciences, Yazd, Iran

**Keywords:** Orbit, Malignancy, Fungal infection, Eye

## Abstract

Fungal infection of the orbit is rare especially among immunocompetent patients. We present a 9-year–old girl with peri-orbital, eyelid and internal canthus swelling of the left eye. Clinical impression was suggestive of malignant tumor such as rhabdomyosarcoma or lymphoma. Histopathological examination of biopsied tissue revealed necrotizing granulomatous inflammation confirmed as fungal infection. Complete response to antifungal therapy was noted after four months.

## INTRODUCTION

Orbital lesions are relatively uncommon and caused by inflammatory and immunological disorders or secondary to systemic diseases such as thyroid ophtalmopathy or less likely due to idiopathic inflammatory pseudo tumors.[1] Orbit is also an organ which may be involved by primary or secondary malignant lesions such as lymphoma, metastatic carcinomas, or neoplasms of adjacent tissues such as conjunctiva, paranasal sinuses, eyelids, or even intracranial neoplasms.[2] Orbital inflammatory lesions are composed of infectious or non-infectious cellulitis. Orbital infectious cellulitis is divided into bacterial, non-bacterial and fungal types.[3] Fungal orbital infection is rare in immunocompetent patients especially in the childhood.[4] We report fungal orbital cellulitis in a 9-year-old immunocompetent female who presented with a large mass like lesion, mimicking neoplastic lesion.

## CASE REPORT

A 9-year-old girl was admitted with large mass like lesion in her left orbital area for a month. There was pain and swelling at the internal canthus and over nasal septum near left orbit. Past medical history showed no allergic rhino-sinusitis, trauma or surgery on her eyes. On inspection no red eye was detected but the mass was firm and had a violet to pinkish colored surface. Her visual acuity was not disturbed and she had no limitation of motion in her left eye. Her mother also pointed out that she used to like and play with vegetables in the garden.

CT scan showed a large mass in the left ethmoidal air cells, left maxillary and frontal sinuses and extending to the medial aspect of extraconal causing mass effect on the left glob to the lateral side and also thinning of the medial bony wall of the left orbit (Fig. 1). Obstruction of the left osteomeatal complex (OMC) was also depicted. The patient underwent biopsy of the lesion for possibility of malignancy. Histopathological examination revealed fibrovascular connective tissue infiltrated by inflammatory cells mostly eosinophils, neutrophils and lymphocytes, with areas of necrosis and granulomatous reaction composed of multinucleated giant cells. Giant cells engulfed fungal hyphae which were thin walled and non–septate that was confirmed by Periodic Acid Schiff (PAS) staining for Zygomycete infection (Fig. 2 A, B, C).

**Figure:**
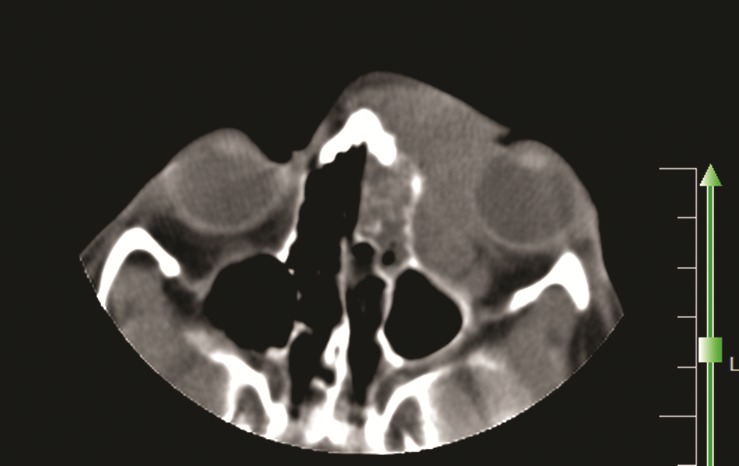
Figure 1: CT scan demonstrated opacity in the left maxillary, ethmoidal air cells, frontal sinuses and on the medial aspect of the extra-conal of the left orbit with moderate mass effect of the lesion on the left glob.

**Figure:**
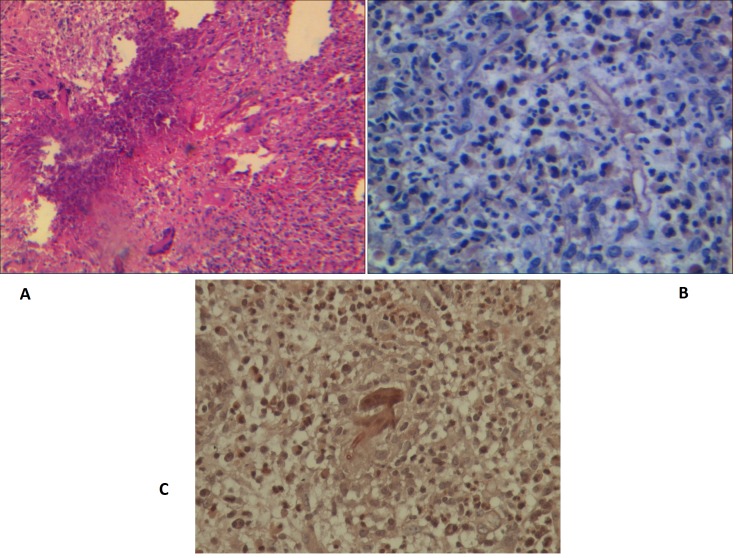
Figure 2: (A) Infiltration of inflammatory cells with areas of necrosis and foreign body reaction(H and E staining ,objective X20). (B) Fungal hyphae inside the fibrovascular connective tissue (PAS staining, objectiveX40). (C) Fungal hyphae inside the multinucleated giant cells (PAS staining, X40).

She was treated with itraconazole, 200 mg twice daily for one week and then 200 mg daily for four other months. After antifungal therapy, the orbital swelling disappeared.

## DISCUSSION

Fungal infections mostly spread from rhino-sinus involvement especially in the patients with history of allergic rhino sinusitis, diabetes mellitus and among those with immunosuppression states such as patients with lymphoma or leukemia undergoing chemotherapy.[3,5] Other routes of orbital fungal infection could be transcutaneous penetration of fungus by trauma or hematogenous spread from primary infected sources such as pulmonary or CNS infection. Direct infection via globe, lacrimal sac, or facial structures has also been reported.[4] No such predisposing factor was found in the index case.

Primary orbital tumors in childhood are mostly, congenital teratoma, lymphoma, rhabdomyosarcoma as a malignant lesions and epidermoid or dermoid cysts, neurofibroma and hemangioma as a benign lesions. Orbital masses have variable presentation such as optic neuritis, sub-periosteal abscess, optic neuropathy, orbital apex syndrome, ophtalmoplegia, epiphora and Duane syndrome.[6] Zafar et al reported fungal orbital infection in an 8-year-old male after non-penetrating trauma to the eye, but our case had no history of trauma to sinuses or orbit. CT scan and MRI are best imaging modalities for fungal infections to describe the soft tissue involvement and extension of inflammation via sinuses or other organs near the orbit.[7] To conclude, rare infections can mimic malignant lesions and effort should be made to diagnose them early.

## Footnotes

**Source of Support:** Nil

**Conflict of Interest:** None declared

